# Exploring how biobanks communicate the possibility of commercial access and its associated benefits and risks in participant documents

**DOI:** 10.1186/s12910-022-00829-1

**Published:** 2022-09-21

**Authors:** G. Samuel, F. Hardcastle, R. Broekstra, A. Lucassen

**Affiliations:** 1 Wellcome Trust Centre for Human Genetics and Centre for Personalised Medicine, University of Oxford, Oxford, OX3 7BN UK; 2 Clinical Ethics, Law and Society Research group, Faculty of Medicine, and Southampton NIHR Biomedical Research Centre., University of Southampton, Tremona Road, Southampton, SO16 6YD UK; 3 Department of Health Sciences, Section Health Psychology, University of Groningen, University Medical Center Groningen of Groningen, Antonius Deusinglaan 1, 9713 AV Groningen, the Netherlands

**Keywords:** Biobanking, health research data repositories, Data access consent, Recruitment, Ethics, Public private relationships

## Abstract

**Background:**

Biobanks and biomedical research data repositories collect their samples and associated data from volunteer participants. Their aims are to facilitate biomedical research and improve health, and they are framed in terms of contributing to the public good. Biobank resources may be accessible to researchers with commercial motivations, for example, researchers in pharmaceutical companies who may utilise the data to develop new clinical therapeutics and pharmaceutical drugs. Studies exploring citizen perceptions of public/private interactions associated with large health data repositories/biobanks indicate that there are sensitivities around public/private and/or non-profit/profit relationships and international sample and data sharing. Less work has explored how biobanks communicate their public/private partnerships to the public or to their potential research participants.

**Methods:**

We explored how a biobank’s aims, benefits and risks, and private/public relationships have been framed in public facing recruitment documents (consent forms and participant information sheets).

**Results:**

Biobank documents often communicate their commercial access arrangements but not the detail about what these interactions would entail, and how risks and benefits would be distributed to the public.

**Conclusion:**

We argue that this leads to a polarised discourse between public and private entities and/or activities, and fails to attend to the blurred lines between them. This results in a lack of attention to more important issues such as how risks and benefits in general are distributed to the public. We call for a nuanced approach that can contribute to the much-needed dialogue in this space.

## Introduction

Biobanks^1^ and biomedical research data repositories collect their samples and associated data from volunteer participants to facilitate biomedical research and improve health, and are framed in terms of contributing to the public good [2–5]. Biobank resources may be accessible to researchers with commercial motivations, for example, to utilise the data for developing new clinical therapeutics and pharmaceutical drugs development [5–8]. For some, this has resulted in concerns over how the benefits and risks of biobanking are distributed [9] and whether biobanks contribute to the common good and/or to private interests [4, 8, 10, 11]. One particular concern is that a commercial company may profit financially from their access to biobank resources, but the high-cost medicine produced as a result may prevent individuals from accessing these therapeutic benefits [4, 12].

Studies exploring citizen perceptions of public/private interactions associated with large health data repositories/biobanks indicate that there are sensitivities around public/private and/or non-profit/profit relationships and international data sharing [13–16]. In particular, there are concerns about the lack of clarity over how benefits are distributed to the public^2^ and to local communities.^3^ There are also concerns associated with data misuse [12]. Nevertheless, collaboration between research organisations and commercial entities is often a necessity for healthcare innovation [4, 12, 18]. On the one hand, a lack of such collaboration can, for example, stifle the drug development pipeline [12]. On the other hand, commercial enterprise needs public research infrastructure and resources [19] since many academic institutions and non-profit organisations are the source of fundamental work that leads to the development of drug products. Publicly funded research is often the precursor for drug-related publications and patents [18]. While the debate about access to biobank resources highlights legitimate concerns, it also often over-simplifies the interdependencies of public–private relations in contemporary societies, especially in health research and health care domain.

Much literature discusses the issues and complexities around public/private partnerships in health databases and biobanking (for example, see [20]), however, less work has explored how biobanks communicate their public/private partnerships to the public, and particularly to their potential research participants. We explored how a biobank’s private/public relationships—in particular, those associated with the ability of commercial companies to access a biobank’s resource—have been framed to potential participants in public facing recruitment documents (consent forms and participant information sheets), within the explanation about the biobank’s aim, purpose and potential benefits and risks. This is important because when reading recruitment documents, participants will potentially view statements about commercial interaction within discourses associated with said biobank’s aims, risks and benefits, with the latter potentially affecting perceptions of the former. We narrowed our research to European biobanks. Our research question was: how do European biobanks communicate information about their purpose, their benefits and risks, and about commercial/industry sector access to their resource to potential participants in their recruitment documents?

## Methods

We limited our search to publicly funded^4^ biobanks recruiting members of the general population (in contrast to those focussing on disease categories).

### Data collection

Our chosen biobanks were sourced from a list published by Gille, Vayena, and Blasimme (2020). The list contained 47 European-based biobanks. We collected the consent form and participant information sheet provided to participants, where available, from each biobank’s website. If the consent forms and participant information sheets were not present on the website, or were not written in English, German, or Dutch (the languages spoken by the authors), we approached the biobank via email to ask for a copy of the documents. 21 national BBMRI (Biobanking and Biomolecular Resources Research Infrastructure)^5^ nodes were also invited, by email, to distribute our invitation email to biobanks within their own jurisdictions. After two follow up emails to non-responders, in total, 19 biobanks were included in our analysis (Table 1).

**Table 1:** Biobanks included in our analysis

Country	Biobank	Country	Biobank
1. Austria	Graz Biobank	10. Netherlands	Radboud UMC biobank
2. Estonia	Estonian Biobank	11. Netherlands	Amsterdam UMC biobank
3. Germany	BioMaterialBank Heidelberg	12. Netherlands	Groningen UMC biobank
4. Finland	Finnish Red Cross Blood Service Biobank	13. Norway	Hunt Biobank
5. Finland	THL Biobank	14. Poland	Wroclaw Research Centre EIT + Biobank
6. Finland	Finnish Clinical Biobank Tampere	15. Sweden	Lifegene
7. Finland	Helsinki Biobank	16. Sweden	VIP/NSHDS (Umeå)
8. Lithuania	IMI Biobank	17. UK	Human Developmental Biology Resource
9. Netherlands	Lifelines biobank and cohort study	18. UK	UK Biobank
		19. UK	Generation Scotland

### Analysis

Authors read consent forms and participant information sheets and discussed their findings and interpretations in online meetings. A top-down coding sheet was developed with five categories that pertained to information on (a) the purpose of the biobank, (b) the benefits that would come from biobank endeavours, (c) commercial interactions, (d) the mention of risk sharing, and (e) any other information provided that stood out. Once information was extracted into relevant categories, information in each category was qualitatively analysed for similarities and differences.

Google translate software and DeepL software were used to translate recruitment documents when required. If specific language/sentences required translating accurately, native speakers were asked for assistance, and this is indicated in the findings.

### Limitations

We recognise that our translation of some documents into English may have lost some clarity of the original. Furthermore, we limited this study to documents from public biobanks, and did not take commercial biobanks into consideration. It will be important to compare framing and articulation of private biobanks in future research to better understand their context [21]. We also recognise that face-to-face discussions at the time of consent may have supplemented written information, and that potential participants may (also) have acquired some information via biobank websites or social media networks. Our exploration excluded any communications on the biobank’s websites, as these were not consistent between biobanks and also evolve/change at different tempos.

## Findings

Our findings highlighted many similarities between the different biobanks’ documents, yet also indicated several key differences. We discuss these findings in three sections—communication about (a) the purpose of the biobank; (b) the benefits associated with participation; and (c) commercial interactions (including risks); also see Table 2.

**Table 2:** Coding sheet of findings

Country	Biobank	Mention commercial interaction?*	Benefits articulated, and processes to ensure distribution of benefit	Mention and framing of risk sharing**
1. Austria	Graz biobank	Yes—states that research results can further be utilized scientifically or commercially, for example for patenting	General population benefit in terms of research and health benefits: ‘Your medical data and samples should lead to scientific knowledge of the causes, course, treatment and prevention of diseases and are eventually used for development and quality control of new diagnostic tools and methods, as well as for the training of health care professionals’	Documents mention that risks are very low, and it is the biobank’s responsibility to ensure data security
2. Estonia	Estonian biobank	Yes—states that the Genebank enables scientific and applied gene and health research	General population benefit in terms of research and health benefits, including personalised medicine. Plus: ‘Donors have right to be aware of their genetic data and other data about me stored in the Gene Bank, except my genealogy, and to genetic counselling upon accessing my data stored in the Gene Bank free of charge’	Documents state how the rights of the donor in relation to prevention of harm are derived from The Human Genes Research Act
3. Germany	BioMaterialBank Heidelberg	No—distinction was made in the PI sheet between whether those researchers who were permitted access were commercial or academic based	General population benefit in terms of research and health benefits ‘The donation of your biomaterial has no personal benefit for you at first. Financial compensation cannot and must not be for ethical and legal reasons. You can help to improve the diagnosis of cancer, infectious diseases, cardiovascular diagnosis of cancer, infectious diseases, cardiovascular diseases or other diseases or other serious illnesses and to research new treatment options’	Documents mention the biobank’s responsibility to ensure data security and an ethical review process. ‘Applications are carefully reviewed by the BMBH and the responsible ethics committee’
4. Finland	Finnish Red Cross Blood Service Biobank	Yes—states that the biobank’s resources can be used by commercial entities as per the Finnish Biobanking Act	General population benefit in terms of research and health benefits, including personalised medicine ‘Research results will be returned to the Biobank for use in future studies’	Documents state that risks of data security breaches are very low
5. Finland	THL Biobank	Yes—states that the biobank’s collaborative projects may result in commercial applications, and this is in line with the Finnish Biobanking Act	General population benefit in terms of research and health benefits, including personalised medicine. Benefits are framed as long-term and in the future due to the research nature of the biobank	Documents mention that the risk is very low, and it is the biobank’s responsibility to ensure data security
6. Finland	Finnish Clinical Biobank Tampere	No—does not mention anything about this topic on PI sheet/consent form	Benefits not mentioned. Documents state that when a study has ended, the results obtained from samples are returned to the biobank for use in future studies	Documents state that the risk of samples and data being misused is extremely low
7. Finland	Helsinki biobank	Yes—the website contained a justification for interacting with pharmaceutical companies	General population benefit in terms of research and health benefits, including personalised medicine. Documents state: ‘you also have the right to request any information about your health status that has been obtained in biobank research. However, it is seldom possible to benefit from the biobank’s research results directly in your own healthcare. If you wish, you can request analysis over the meaning of the results analysed from your samples, but a fee may be charged to cover the costs of the result verification and analysis’	Documents state that appropriate prerequisites for research are assessed in advance, so the risk of the samples and information in the biobank being misused is very low
8. Lithuania	IMI Biobank	Yes—provides a short justification of why it is important to provide access to pharmaceutical companies	General population benefit in terms of research and health benefits, including personalised medicine. Furthermore, the documents state: ‘You yourself have the right to actively contact the IMI Biobank and receive information on whether your biological sample and health information has been used. You will be able to get information about a specific biomedical research and its goals, as well as the results obtained’ This could be interpreted as relating to benefit sharing or to the right to information	Documents state the biobank will make every effort to ensure that data security is not violated. They also state that they have civil liability insurance for property and non-property damage that can ensure that those participating in the activities of the biobank are remunerated
9. Netherlands	Lifelines biobank and cohort study	No—does not mention commercial interactions, but explains that inventions might lead to intellectual property in PI sheet	General population benefit in terms of research and health benefits, including personalised medicine. No direct individual benefit bar via incidental findings. Though general population benefit was framed as an in-direct individual benefit. As such, individual benefit was viewed as a long-term outcome of biobank related research in terms of improved future health care	Documents state that there is little risk and research will be conducted confidentially and in line with data protection legislation. Department of legal affairs and insurance companies are excluded from accessing the data
10. Netherlands	Radboud UMC biobank	Yes—explains in PI sheet the importance of collaboration for some research projects, as well as potential consequences for intellectual property and profit development, though stipulating that all benefit healthcare	General population benefit in terms of research and health benefits, including personalised medicine. Specifically, benefits are articulated as gaining knowledge, which benefits certain patient groups. Actionable incidental findings will also be communicated	Documents provide an explanation about data protection and privacy, especially if data is sent to non-EU countries
11.Netherlands	Amsterdam UMC biobank	Yes—the PI sheet explains in detail the consequences and process of collaboration, especially about responsibilities and conditions. The consent form offers and option for non contribution to these research projects	General population benefit in terms of research and health benefits, including personalised medicine. No direct individual benefit bar via incidental findings and improved future health care benefitting individuals indirectly	Documents mention disadvantages/risks of taking biosamples, and articulate no to little risk regarding privacy concerns
12.Netherlands	Groningen UMC biobank	Yes—the PI sheet explains reasons, consequences and the process of collaboration, especially about responsibilities and conditions	General population benefit, with knowledge gain in health and illness coming primarily from scientific research	Documents provide an explanation about data protection and privacy, especially if data is sent to non-EU countries
13. Norway	Hunt	Yes—the biobank provides detailed information on why it is important to provide access to commercial entities	General population benefit in terms of research and health benefits, including personalised medicine. Documents state that there is no direct individual benefit bar feedback such as activity measurement and blood test results from data collection. Incidental findings are also returned, if relevant	Documents mention that data security is managed via IT solutions and strict data management
14. Poland	Wroclaw research centre EIT + biobank	Yes—states that they cooperate with commercial ventures	General population health is mentioned. Incidental findings with clinical relevance will be communicated. The document also states: ‘As part of the project, patients participating in it will be guaranteed a free panel of blood tests (levels of homocysteine, ultra-sensitive CRP, glucose, insulin, total cholesterol, triglycerides, HDL, LDL). These results will be used to better characterize the health condition of the inhabitants of the voivodship, and the results will be sent to you via Polish Post, by registered letter with acknowledgment of receipt.’	Documents state that the biobank will take all appropriate technical measures to protect personal data
15. Sweden	Lifegene	No—the biobank does not allow commercial researchers access to the biobank resource	General population benefit in terms of research and health benefits: 'In the long run, we want to be able to prevent, diagnose and treat diseases such as allergies, depression, infections, cardiovascular disease and cancer'. The consent form states: ‘You have the right to once a year, free of charge, know what information that is registered in LifeGene about you, from where information has been downloaded and to whom the data may have been submitted. An excerpt showing this information can be obtained after a signed request made to LifeGene. If it turns out that any information is incorrect, you have the right to have that information corrected, blocked or deleted’	Documents do not mention any risks due to participation. They do state how data will be kept secure
16. Sweden	VIP/NSHDS (Umeå)	Yes—thee biobank is in collaboration with a commercial partner and they explain this-and how the resource will be used-in the PI sheet and consent form	General population benefit in terms of research and health benefits, including personalised medicine. No direct individual benefit bar via incidental findings. Population benefit framed in terms of a regional benefit, but not described in any detail	No
17. UK	Human Developmental Biology Resource***	Yes—states that research may be carried out in commercial entities	General population benefit mentioned in terms of research and health benefits. PI sheet mentions: ‘What are the possible benefits of taking part? The donated tissue may be used in a number of research projects which will hopefully lead to a greater understanding of human development and ways in which we can identify and treat genetic diseases. The results of the research may contribute to the development of new drugs and treatments. However, you would not benefit from or be entitled to any profits resulting from this work’	The PI sheet states: ‘What are the possible risks of taking part? There are no additional risks. The treatment before, during, and after your procedure will be the same whether you decide to donate or not. and …because your genetic information is unique to you, there is a very small chance that someone could trace it back to you, by matching your genetic information to other entries on the internet. The risk of this happening is extremely small, but may grow in the future.’
18. UK	UK Biobank	Yes—the consent form requires a participant to declare that they will not benefit financially from taking part (e.g. if research leads to commercial development of a new treatment). The PI sheet, states in several places that commercial companies will be able to access the bioresource for approved research	Consent form mentions that participants will not receive financial benefits and frames future generations as main recipients of benefit. The consent form has a section stating: 'I understand that none of my results will be given to me (except for some measurements during this visit) and that I will not benefit financially from taking part (e.g. if research leads to commercial development of a new treatment)'. The PI sheet mentions: 'Like giving blood for transfusions, UK Biobank is not intended to help directly those who take part—but it should give future generations a much better chance of living their lives free of diseases that disable and kill'	PI sheet states:'the risks of participants suffering harm as a result of taking part are minimal, and UK Biobank has insurance in place to provide compensation for any negligent harm caused by participation'. The PI sheet also states: 'Taking part in UK Biobank should not cause you any harm. The project aims to observe what happens to participants over the next few decades so that future generations can benefit. It is not intended to change directly what happens to people who take part: in particular, the initial assessment visit is not a “health check”. Apart from providing you with the results of some standard measurements made during that visit, none of your results will be given to you or your doctors (even if the results do not seem to be normal). This is because such feedback outside of the normal clinical setting is of questionable value, and might even be harmful (for example, causing undue alarm and having potentially adverse effects on insurance status), especially when given without prior counselling or support... Participation involves a minimal risk in relation to the use of personal information. Great care will be taken to ensure the confidentiality of all data (see below) and the risk to participants of a breach of confidentiality is considered very low." In a different section about potential risks the PI sheet states:" Over the coming years, a very wide range of tests will be done on your blood, saliva and urine samples for approved medical and other health-related research. Details that might identify you will be removed from any information and samples provided to researchers in order that they cannot be traced back to you. None of your particular test results will be fed back to you, your doctors or anyone else. So, taking part should not have any adverse effects on you (including your employment status or ability to get insurance)'
19. UK	Generation Scotland	Yes—the consent form requires the participant to declare that they understand that 'the Universities and funders involved in this project have a financial interest in using the results to develop new treatments and that this may involve commercial companies (e.g. drug companies)'. The PI sheet elaborates further on the subject of commercialisation and has a section on 'What about commercialisation? Will industry be involved in any way?'*“*	The PI sheet explicitly has a question about comemrcialisation in which they expand on benefit sharing. A part of this states: "This project is designed first and foremost to further public good….Any health-related benefits of this work may take many years to develop and successful developments may give rise to intellectual property rights, such as patents. Individual contributions do not have any financial value by themselves and you will not receive any financial gain from taking part. However, some of the revenue from any successful commercial projects will be returned to support the NHS and health research" Also, the consent form states: I understand that ". The Universities and funders involved in this project have a financial interest in using the results to develop new treatments and that this may involve commercial companies (e.g. drug companies) . I am giving blood as a gift and I will not have any legal claim on it . I am giving consent for my blood and materials prepared from it (e.g. DNA) to be used for genetic studies. The results of these studies may be used for making patent applications, without any payment to me or my family"	The PI sheet only mentions disadvantages /risks to taking part in terms of the risks of sampling blood from the potential participant

*Commercial interaction here only referred to commercial access to a biobank’s resource, rather than with regard to a biobank’s operational management. Note: this information does not tell us whether (or not) biobanks interact with commercial companies, only whether it was stated in the recruitment documents

**Risk is all risk associated with participation, excluding acute risk from the physical action of taking biosamples or completing health questionnaires

***We were only able to obtain the PI sheet for this biobank, and not the consent form

### Purpose of biobanks

Within the recruitment documents, nearly all biobanks framed their purpose as supporting health research to promote a better understanding, or prevention, of health conditions and/or disease, and/or to develop new diagnosis or treatment options. Several biobanks provided more detail about the types of diseases that would be studied using the samples and associated data. This included common diseases that have widespread effects, including heart disease, stroke, dementia, diabetes, and cancer: ‘to improve the diagnosis of cancer, infectious diseases, cardiovascular diseases or other serious diseases’ (Heidelberg, Germany); ‘such as cancer, heart disease, diabetes, dementia, and joint problems’ (UK Biobank).

Biobanks used different language to describe the possibility of health benefits accruing from any biobank associated research. Most biobanks described the possibility of health benefits using conditional language, or by pointing to the fact that research takes time and participants are unlikely to see (m)any of these benefits in the near term (THL Finland, Generation Scotland). Phrases included, for example, health research ‘will hopefully lead to’ (HDBR UCL). Some biobanks used more optimistic language, pointing to positive expectations about future benefit in their claims. The UK Biobank participant information supplement explained that because the venture involved thousands of people:

‘it should be able to show more reliably than ever before why some people develop that disease while others do not. This should help to find new ways to prevent death and disability from many different conditions’ (our underline, UKB Further information leaflet pp.4).

Finally, the language used in some biobank documents promoted the importance of biobank associated health research as a key factor for improving health. For example, the Austrian biobank’s material described the health research that biobanks supported as: ‘one of the most important requirements for a better understanding of the causes and courses of diseases, and for the development of new methods for diagnosis, prevention and treatment of these diseases (translated by native speaker). Lifegenes recruitment documents similarly stated that the biobank: ‘welcome [individuals] to join the fight for one healthier Sweden’.

### How benefits and benefit sharing is framed and articulated

The majority of the recruitment documents emphasised that participants were unlikely to see any individual health benefits from research supported by the biobank: ‘biobank research does not yield results for individual donors’ (AMC NL). Some biobanks also stressed that no financial benefit would come from participation: ‘I may not demand a fee for providing a tissue sample’ (Estonian Biobank). Discourses of benefit, instead, were framed in general statements about the likely benefit to the population as a whole, both now, and for ‘the society of the future’ (IMI, Lithuania[googletranslate]). Umeå’s (Sweden) documents framed benefits in terms of regionalistic (cf nationalistic) discourses rather than benefit at the general population level.

In our region, there are many families with diseases for which there has been no treatment to-date. In the future, it may be possible to get help for both these diseases and major public diseases….With UmanGenomics [Umeå biobank], the value of the discoveries made here can also benefit our region.

The exception to this was that some biobanks provided a choice to participants about whether they wished to receive additional or incidental findings (depending on the biobank). This choice was often framed in different ways. For example, in the Amsterdam Medical Centre biobank documents, the decision to provide an individual with an incidental finding was ultimately at the discretion of a clinical practitioner, who would weigh up the risks and benefits associated with passing on such knowledge:

[regarding incidental findings] your general practitioner….will consider whether it is necessary to inform you of such a finding….Criteria that play a role [in this decision] are the seriousness of the possible consequences for your health and that of your immediate family and the treatment options….If…an incidental finding is reported to you, this may have consequences for insurance and medical examinations…

The Estonian biobank, on the other hand, framed the decision in terms of rights. The recruitment document emphasised the various rights participants would have to access information about themselves, as well as the right to access genetic counselling services in the event of accessed findings:

I have the right not to be aware of my genetic data, hereditary characteristic and genetic risks obtained as a results of genetic research…I have the right to be aware of my genetic data and other data about me stored in the Gene Bank, except my genealogy. I have the right to genetic counselling upon accessing my data stored in the Gene Bank. I can access my data stored in the Gene Bank free of charge.

Hunt biobank, in contrast, framed the decision to receive information about feedback on risks of preventable genetic diseases as nudging: ‘you make an important contribution to health research, and can get interesting and useful information about your own health’ (Hunt biobank).

Finally, in some recruitment documents, information on the distribution of benefits was discussed in terms of being distributed to the biobank. In this way, the biobank was constructed as a steward for public and participant benefit sharing: ‘when a study [conducted by a researcher who is using data from the biobank] has ended, the results obtained from samples are returned to the biobank for use in future studies’ (Tampere, Finland). For Generation Scotland, not only was the biobank constructed as the recipient for these benefits, but so was the UK NHS, a trusted public institution [22]: ‘some of the revenue from any successful commercial projects will be returned to support the NHS and health research’. On the other hand, Lifelines (NL) explained that benefits would emerge from the publication of findings in scientific journals, which ‘will improve health care in general and thus provide individual benefit. (Lifelines NL).

### Direct mention of commercial interactions

A minority of biobanks explicitly stated that their resource could not be accessed by researchers working for commercial entities. For the remainder, many—though not all the biobank documents—explicitly stated that there would be commercial access to the biobank’s resource. Some documents simply noted the possibility of a commercial interaction. Others provided information on the role of commercial access–a role that was framed in terms of potential development of new medications or treatments:

research may also take place in cooperation with companies, such as for the production of medication (VIP/NSHDS (Umeå).

Some documents explicitly tried to justify commercial entity interactions as a necessity for the drug development process: ‘pharmaceutical companies fund for example, lung cancer, melanoma research using biobanks, and health information from biobanks, to find new drugs’ (IMI, Lithuania [googletranslate]).

Two biobanks’ documents—THL and the Estonian Biobank—used a legal justification to warrant interaction with commercial entities. The counties within which both biobanks are situated have a specific Biobank Law to regulate biobank activities and processes:

The samples and the related data can be used in various research projects, and in commercial cooperation and product development projects even outside the European Union, as permitted by law. (Finnish Blood Biobank);

The Human Genes Research Act regulates the rights of gene donors. […] This consent form, the law, and information kit shall be explained to me … the Gene Bank enables scientific an applied gene and health research […] I am aware that my tissue sample may have some commercial value and research and development institutions as well as commercial enterprises may receive anonymous data about gene donors. (Estonian Biobank).

Finally, a handful of biobanks dedicated sub-sections of their recruitment documents to provide information about their interaction with the commercial sector. For example, Hunt biobank’s section: ‘Why does Hunt wish to collaborate with (health) industries?’, and Generation Scotland’s section: ‘What about commercialisation?’.^6^

The commercial sector was sometimes just mentioned, with no attempt to define or distinguish between types of commercial access to the biobank resource. Other times, more detail was provided about the commercial organisation, for example, that it was an approved organisation^7^ (‘the research is carried out in approved research organisations. These may be in the public or private sectors’ (UK Human Developmental Biology Resource)); that they needed to collaborate with someone affiliated with a university (‘Research collaboration with the health industry will be leaded by someone affiliated with a Norwegian university’ (HUNT biobank); or that it was research departments specifically at pharmaceutical and diagnostic companies that would be conducting the research (‘biobanks cooperate primarily with research teams at universities, research institutes and hospitals as well as with research departments of pharmaceutical and diagnostic companies’) (Wroclaw Research Centre EIT + Biobank, Poland [google translate]).

Statements about commercial access were often followed by descriptions of the strict data governance regulations the biobank would adhere to, to ensure that participant samples and data were protected, and that data would only be given to commercial entities that had met strict requirements for access:

Research may also take place in cooperation with companies…Before the samples may be used, the Medical Biobank’s experts review the research project’s purpose and scientific value. A regional research ethics committee conducts an independent evaluation and decides if the research is ethically acceptable (VIP/NSHDS (Umeå))

Statements about data security in relation to commercial research were also utilised to emphasise security more broadly in many of the biobank documents. For example, many biobank documents stated they would ensure anonymity of samples and data, and that participation conferred generally low or no risk. While some biobanks described what risks could occur in more detail (for example, data misuse or data breaches), others focused on how these were prevented by data management or IT-solutions. Some biobanks articulated legal limitations for data use or their liability for harm due to participation, for example by insurance coverage. No other notions of risk were discussed (bar those associated with the physical risk of having blood removed, where relevant) (see Table 2).

## Discussion

We were interested in exploring how European biobanks’ private/public relationships have been articulated to potential biobank participants in recruitment documents within the wider explanation about the biobank’s aims, purpose, as well as potential benefits and risks. Our analysis illustrated that recruitment documents frame health, and knowledge about health and illness, as important; and that biobank supported health research was a key approach to achieving this. Health benefits were described in recruitment documents at the national population (or sometimes regional) level, and as a ‘common’ benefit of more or better diagnostics and treatments for diseases—especially well-established, wide-spread diseases (for example, see [2]). Some biobanks articulated how benefit would be distributed, especially with an individual, though at the general level, there was little explanation of how these benefits would be developed.

While biobank recruitment documents articulated participant benefits at a general level, they separated academic researchers and commercial enterprises. This separation often amounted to a statement about the fact that commercial entities were permitted to access samples/data, though some documents justified this access as a necessary step to achieve the overall aim of improving health and health care. A minority stated that biobank participants would not gain from any private-sector profits. There was little further description of what such relationships looked like.

Merely stating the presence of commercial-biobank interactions may suffice for some participants to be able to make a decision about whether to participate in a biobank [23, 24]. Furthermore, it may reflect the fact that providing further information may be difficult to do in the complex and changing nature of public-private sector relationships. However, another hypothesis could be that the lack of information provided about commercial-biobank interactions—and, in particular, providing too simple a separation of commercial versus non-commercial—could amplify reductive thinking that classifies public and private sectors as polarised opposites of morally good and bad. Such thinking ignores the complex relationship between public and private sectors, as well as the fact that research activities from both the public and private sector may be considered more or less problematic. For instance, if an academic researcher uses their research to develop a spin-off company it may be viewed as more problematic than if a commercial company develops a diagnostic test that is equitably distributed. Such missing narratives provide a vacuum for other discourses and ideas to fill. Missing narratives could therefore heighten scepticism of those who are already worried about biobank-commercial interactions. This could lead to non-participation,^8^ or a breakdown in trust between potential participants and biobanks, or could lead to participants signing-up, but feeling resigned to, rather than comfortable with commercial involvement. Such resignation associated with consent has been identified in some of our own unpublished interview findings in this and other fields, including for participants of the UK 100,000 genomes project, for members of the public using the COVID-19 UK NHS app, and during social media data use consent processes. Here, individuals have commented that they have made the decision to consent to their data being used by an institution or technology even though they remain uncomfortable with commercial access to their data. This is because they feel a lack of empowerment to be able to question commercial involvement or alter it; rather viewing it as part of contemporary society and something they have little control over.

We appreciate that most consent processes involve a discussion that may be in addition to/ not reflected in the recruitment documents. Discussions during the consent process may have provided a useful avenue for further details about commercial involvement to be communicated to participants. We also appreciate that the consent process (and associated paperwork) cannot do all the ‘ethical work’ for ensuring such details are communicated [25]. In fact, in the process of our research, we found that some biobank websites provided more information about commercial interactions than the detail supplied in their recruitment documents, highlighting how ethical work goes beyond the consent and recruitment phase. Some of these websites also permitted participants to view access agreement policies between the biobank and commercial entities. Here, more detail was often provided about biobank-commercial interactions, including that a commercial company may not sell a biobank resource, but that they may profit from such access. In fact, the temporal nature of such interactions means that websites offer a useful approach to ensuring transparency and open communication about the benefits and risks associated with biobank-associated research, and can provide more detail about commercial interactions. For example—especially for those biobanks whose recruitment has now ended or where recruitment documents are fixed—websites can portray an up-to-date and appropriate sense of the uncertain futures from biobank associated research. They can also document that participation in health research is only one way to contribute to improved health; contribution to social factors, such as better education, better housing, more job security, and less poverty are other important determinants of improved health.^9^

While previous research suggests that most biobanks aim to be transparent about their governance mechanisms on their websites, it has also emphasised that these biobanks present little detail on accountability and oversight mechanisms [26]. We argue that biobanks should provide detail about the nature of their public/private interactions, as well as the benefits and risks involved in these interactions, on their websites, so that these platforms can do some of the ethical work alongside recruitment documents [27–29]. This can redress the fact that potential biobank participants do not always adequately attend to information about data sharing during the consent or participation process, rather relying on generalized trust instead of closely reviewing such disclosures being fully informed [30–34].

At the same time, websites are not a technical fix to addressing concerns about public–private interactions in biobanking for two reasons. First, websites may only be seen by some participants, meaning that many individuals will participate without reviewing this material. Various approaches could lead more participants to review the information on a website, possibly by informing them about its availability and encouraging them to review it during the consent process or at another point. Sending regular newsletters or other outreach to participants via different media channels are other options that are already applied by several biobanks, though the same representation biases might apply for these communication channels too.

Second, providing solutions about how best to present information about public–private interactions ignores the need to not only explain public–private interactions in a more nuanced way, but to also assess the benefits and risks associated with such interactions with more nuance. Public–private relationships are much more complex than an articulation of distributed risks, harms and benefits and public dialogue is one way to ensure the nuances of public/private sector interactions are properly understood and addressed in a way that considers both biobank participants, as well as society more broadly. Public dialogue needs to go further than engaging the public broadly about their views on the topic. This is because such engagement lacks incentive for biobanks to address any concerns raised and/or could lead biobanks to addressing any concerns in their own way. For example, the inadequate descriptions of public–private interactions provided in some biobank recruitment and consent forms that we analysed is perhaps a consequence of biobanks responding to public concern about these interactions in their own way. Better is to ensure that public dialogue is central to the governance of a biobank. Koenig [35] has proposed that lay people should review aspects of biobank governance through participation on oversight boards, rather than being asked to review such information individually. Samuel and Lucassen [36], too, have argued that participants should be a central aspect of biobank committees, where they should be involved (more or less) in decision-making associated with granting access to a biobank’s (sample or) data resource. A good example of this working in practice is at Genomics England. Genomics England runs the UK’s 100,000 genomes project, which sequenced 100,000 genomes from UK National Health Service patients for both clinical care and research. The committee that provides commercial access to this resource is comprised of a number of participants, who play a crucial role in decision-making, including an ex-post review after access to ensure that any access leads to benefits for all (*forthcoming*).

Overall, instead of viewing public/private interactions as binary, it might be more helpful to show how contracts can be formed where the benefits of new technological/scientific advances and innovations are reclaimed for the common good and do not just serve the interests of a few [9]. This builds on the interpretation of public biobanking as a new form of social contract, which requires the interest of a population to be protected with a systematic and group level approach rather than being primarily incidental and individual-based [9]. Taking this lead, a more helpful way for biobanks to address citizens’ concerns about their involvements with commercial actors may be to detail with different tools and procedures how commercial actors would share risks and benefits with the public and how the benefits would be returned to the local communities. This leaves room for biobanks to adapt to their relevant contexts. Biobanks’ current communication strategy in their recruitment forms seems to send contradictory messages. On the one hand, the aims of biobanks are communicated as an ethos of public good. On the other hand, risks are individually framed in terms of concerns about privacy and data governance; and benefit sharing is primary documented through individually based incidental and/or additional findings. More of a focus on collective benefits and risks to communities can pave the way to a more nuanced discussion.

Concluding, the biobank documents we analysed were transparent about their commercial interactions, but lacked detail about what these interactions might entail, and what would be the distribution of benefits and risks. We appreciate this is a difficult balance for biobank literature to get right: the changing nature of commercial interactions with advances in technologies, for example, means that explanations at the time of recruitment may have evolved. Providing too simple a separation of commercial versus non-commercial however, runs the risk of establishing or perpetuating a polarised and unhelpful discourse that views public/private sector interactions are necessarily problematic. We are not advocating a change in the consent process, but instead proposing more transparency by presentation of key information in other places and supporting public engagement on the topic. Public engagement can help build trust. Those involved in the process can also help shape the terms and conditions for biobanks’ public–private interactions. Moreover, they can assist biobanks understand how best to articulate public–private interactions in public-facing material so that the nuances of public/private sector interactions can be more explicit in terms of how private entities will share risks and benefits with the public, and how any benefits should be returned to the local communities.

## Data Availability

The datasets analysed during the current study are available from the corresponding author on request.

## Footnotes

1 We acknowledge that the term ‘biobank’ has various definitions that encapsulate a wide range of practices. However, in general, the term is used in medical research to relate to the collection of biological samples and their associated data from participants [1]. Where previously researchers held their own collections of samples, biobanks harness the increased power to determine significant findings by combining samples and data at scale.

2 DeepMind Technology Limited’s access to data from United Kingdom’s Royal Free London National Health Service (NHS) Foundation Trust to improve care for acute kidney injury, raised concerns about a lack of transparency, and privacy of the data subjects [17].

3 See https://wellcome.org/sites/default/files/public-attitudes-to-commercial-access-to-health-data-summary-wellcome-mar16.pdf

4 We note that public private funding arrangements are complex. We were interested in those biobanks marketing themselves as public sector biobanks established for the ‘common good’.

5 The BBMRI is a distributed research infrastructure of biobanks and biomolecular resources. https://www.bbmri-eric.eu/.

6 On the Helsinki (Finland) Biobank website it states how medicine development is currently exclusively the responsibility of the pharmaceutical industry and that is why it is important that pharmaceutical companies are also represented in the research. We could not find this information in the recruitment documents. https://www.helsinginbiopankki.fi/fi/kysymyksia-helsingin-biopankista

7 Sometimes (though not always) approved research(ers) were defined as those having appropriate ethics approval (for e.g., UK Biobank; Umeå).

8 The recent UK NHS General Practice Data for Planning and Research (GPDPR) proposal to create a centralised database of pseudonymised patient data for researchers and commercial partners is a case in point. Millions of citizens opted out because of the lack of openness, transparency and public engagement.

9 See https://www.who.int/health-topics/social-determinants-of-health#tab=tab_1
